# The Potential of Two Entomopathogenic Fungi and Enhanced Diatomaceous Earth Mixed with Abamectin: A Comprehensive Study on Mortality, Progeny Production, Application Method, and Surface Application against *Tribolium castaneum*

**DOI:** 10.3390/pathogens12060773

**Published:** 2023-05-29

**Authors:** Waqas Wakil, Nickolas G. Kavallieratos, Nikoleta Eleftheriadou, Tahira Riasat, Muhammad Usman Ghazanfar, Khawaja G. Rasool, Mureed Husain, Abdulrahman S. Aldawood

**Affiliations:** 1 Department of Entomology, University of Agriculture, Faisalabad 38040, Pakistan; tahirariasat@yahoo.com; 2 Senckenberg German Entomological Institute, D-15374 Müncheberg, Germany; 3 Laboratory of Agricultural Zoology and Entomology, Department of Crop Science, Agricultural University of Athens, 75 Iera Odos Str., 11855 Athens, Greece; nikolelef@aua.gr; 4 Department of Zoology, GC University, Faisalabad 38000, Pakistan; 5 Department of Plant Pathology, College of Agriculture, Sargodha University, Sargodha 40100, Pakistan; usmanghazanfar1972@gmail.com; 6 Department of Plant Protection, College of Food and Agriculture Sciences, King Saud University, Riyadh 11451, Saudi Arabia; gkhawaja@ksu.edu.sa (K.G.R.); mbukhsh@ksu.edu.sa (M.H.); aldawood@ksu.edu.sa (A.S.A.)

**Keywords:** natural pesticide, biopesticide, treatment, storage pest, integrated management

## Abstract

This study determined the efficacy of *Beauveria bassiana* (Bals. -Criv.) Vuill., *Metarhizium anisopliae* (Metchnikoff) Sorokin, and diatomaceous earth mixed with abamectin (DEA) alone and in their combinations for the integrated management of larvae and adults of *Tribolium castaneum* (Herbst) from three field populations of Pakistan (Multan, Rawalpindi, and Rahim Yar Khan) and one laboratory population (Faisalabad). Treatments were applied on three surfaces, namely, viz. steel, concrete, and jute bags, implementing two application methods, dusting and spraying. The combined treatments were more effective in comparison with single treatments for both larvae and adults. Overall, the highest mortality rates were recorded in the Faisalabad population, followed by the Rehaim Yar Khan, Rawalpindi, and Multan populations. Progeny production was suspended 21 days after exposure to the combined treatment of DEA and both fungi in all populations except Rawalpindi. Larvae were found to be more susceptible than adults in all treatments and intervals. Dusting was more efficient than spraying for both larvae and adults and for all the populations studied. The present study provides a wholistic understanding of the impact of different factors on the success of the combined treatments using DEA and entomopathogenic fungi, supporting their use as surface treatments.

## 1. Introduction

The presence of extraneous matter in stored commodities, such as insect pests and their fragments, filth, and insect excreta, indicate unhygienic practices during the process, production, and storage of products [1], compromising their quality and rendering them unfit for human consumption [2]. Approximately 5–10% of the world’s grain production is lost each year due to insect damage [3]. It is estimated that developing countries such as Pakistan experience storage losses of 20% or higher [4], which translates to a yearly monetary loss of USD 500 million to USD 1 billion [5]. In contrast, developed countries experience storage losses of about 9% [6]. Containing over 250,000 described species, Coleoptera is the largest order of insects [7], which includes 600 notorious stored grain species, causing substantial grain loss [8].

*Tribolium castaneum* (Herbst) (Coleoptera: Tenebrionidae) is one of the common pests of stored grains worldwide [9,10,11]. It is a common pest of wheat flour [12], also infesting beans, barley, dried fruits, corn, oats, legumes, milk chocolate, millet, nuts, cottonseed, peas, rice, sunflower seed, and cacao [13]. Larvae and adults of *T. castaneum* are known to feed on broken grain or grain dust but not on intact grains [14]. However, White et al. consider *T. castaneum* a primary pest, since it was observed effortlessly feeding on intact grains [15,16]. The larval stage of *T. castaneum* is the most destructive, as it feeds on the endosperm of seeds [17]. Feeding, molting, and excretions of *T. castaneum* decrease the weight, nutritional value, and quality of grains [7,10]. Aside from direct damage to the grains, the release of benzoquinone-derived substances from the abdominal glands of *T*. *castaneum* further degrades the quality of stored products [18], which become adulterated, with a pungent odor [19,20]. Adults of this species have a habit to form clusters that create a warm environment in winter months, necessary for their development and survival [21,22]. While the pest status of *T. castaneum* is considered secondary, typically requiring prior infestation by an internal feeder, this species is often very common in the complex of pests that attack stored wheat [11], causing substantial loss in storage due to its high reproductive [23,24,25] and intrinsic rate of increase [26]. In addition, it transmits some pathogenic bacteria and fungi [27,28].

Insecticides, mainly pyrethroids and organophosphates, have long been used as grain protectants for the control of most stored-grain insects [29]. However, the excessive use of several insecticides has led to the development of the resistance of stored-product pests, undermining the effectiveness of management [10,29,30]. It has been documented that *T. castaneum* has acquired resistance to several commonly used insecticides, namely, cypermethrin, bifenthrin, pirimiphos-methyl, and malathion [7,31]. Consequently, it is imperative to employ appropriate safety management strategies to control the propagation of *T. castaneum* and effectively manage its infestation.

Incorporating biopesticides as substitutes for traditional chemical pesticides in the context of IPM (Integrated Pest Management) programs may help to decrease the dose of chemical insecticides, avoiding any deleterious effects on the environment [13,32,33]. In this regard, entomopathogenic fungi (EPF), have demonstrated their potential as biocontrol agents against a wide spectrum of insect pests in storages [34,35]. More than 750 species of fungi are entomopathogens [36]. Several of them have been proposed to be formulated as mycoinsecticides against stored-grain insect pests [37] because of their elevated virulence and low toxicity towards mammals [38,39]. EPF are considered advantageous over conventional insecticides due to their long-term residual persistence [40]. This attribute is desirable because EPF can persist and propagate even on cadavers, thereby introducing more inoculum into the environment [41]. The infection process starts with the attachment of the spores to the insect, followed by the penetration of spores through the insect cuticle by secreting certain cuticle-degrading enzymes [42]. Subsequently, hyphae colonize the body of the insect, releasing mycotoxins, resulting in the death of the target insect pest in a few days [42,43]. The key factors determining the efficiency of the infection include various lytic enzymes and primary and secondary metabolites generated by EPF [44]. EPF of the genera *Beauveria* [45], *Isaria* [46], *Lecanicillium* [37], and *Metarhizium* [47] proved very efficient in the reduction in insect populations.

Anamorphic fungi such as *Beauveria bassiana* (Bals. -Criv.) Vuill. (Hypocreales: Cordycipitaceae) and *Metarhizium anisopliae* (Metchnikoff) Sorokin (Hypocreales: Clavicipitaceae) are potent biocontrol agents, infesting numerous hosts, and produce large numbers of spores over several asexual life cycles [48,49]. *Beauveria bassiana* [50,51,52,53] and *M. anisopliae* [54,55,56] have been extensively investigated for their effectiveness in controlling *T. castaneum.* However, the repeated use of entomopathogenic fungi can lead to the development of resistance in *T. castaneum* populations [57,58]. This phenomenon can be explained by the hypothesis of the Red Queen, which proposes that for evolutionary purposes, the host must continuously adapt to defend against its natural enemies [59]. Thus, the repeated use of the same fungal strain can lead to the selection of individuals with higher levels of resistance, rendering the fungi less effective over time [57]. Insect infection with EPF increases enzyme activity, resulting in their sensitivity to insecticides and, consequently, to insect mortality [60]. This opens possibilities for developing effective combined biological products, implementing EPF [60]. Padín et al. [61] investigated the impact of *B. bassiana* on *T. castaneum*, observing that *B. bassiana* did not exhibit significant control over the grain loss caused by *T. castaneum*. However, the combination of the desiccant pesticide *B. bassiana* and diatomaceous earth (DE) has been exhibited to have an additive effect on the adults of *T. castaneum* [53,62].

Diatomaceous earths (DEs), which are comprised of amorphous silicon dioxide [63], are the fossilized remains of diatoms, a major group of algae [64]. DEs are considered an advantageous alternative to pesticides due to their non-toxic nature to non-target organisms [65,66], and their convenient substraction from the grains prior to the milling proccess [67]. Their mode of action involves the absorption of lipid contents (wax) from the cuticle of the insect, ultimately leading to the insect’s demise [68,69]. A disadvantage of their use is linked with the high rate of application, as this issue can have a notable impact on the bulk density of the grains and the presence of dusty residues [70]. To overpower this limitation, DEs can be combined with other control methods, such as insecticides [71], EPF [32,72,73], and oils [74]. Commercially available DEs are effective against various insect species on different stored grains, but the effectiveness of DEs frequently differs depending on various factors (e.g., formulation, treatment approach, stored product) [75,76]. Employing DEs and EPF in conjunction as grain protectants could reduce the necessary application doses due to their distinct mechanisms of action and impact on the insect cuticle [51]. Furthermore, a blend of EPF and DE could be advantageous as a long-lasting protectant, given that both EPF and DE are persistent on grains [51].

There are several reports on the additive effects of DEs combined with either *B. bassiana* [77,78] or *M. anisopliae* [55,79] against *T*. *castaneum.* Nevertheless, the combination of DEs with both *B. bassiana* and *M. anisopliae* has not yet been studied. Therefore, the objective of this study is to explore the effects of the combined formulations of DE and EPF, *B. bassiana* and *M. anisopliae*, the application method, and the treated surface on the mortality and offspring production of *T. castaneum* populations under laboratory conditions, with the aim of simulating the real-world application scenarios employed for the preservation of hard wheat [80]. This study aims to provide useful insights into the potential use of this combination as a combined biological/natural product for the sustainable management of *T. castaneum*.

## 2. Materials and Methods

### 2.1. Insect Culture

Field populations of *T. castaneum* were collected from three sites of Pakistan, i.e., Rahim Yar Khan, Multan, and Rawalpindi. One laboratory population from Faisalabad (Pakistan) was reared for >10 years in the Microbial Control Laboratory, University of Agriculture, Faisalabad, Pakistan. This population had not been subjected to any chemicals, including phosphine. The culture has been conducted on wheat flour +5% brewer’s yeast by weight at 30 °C and 65% RH in 0:100 (Light: Dark) [81].

### 2.2. Grains

Clean, non-infested, and contamination-free wheat, *Triticum aestivum* L. (var. Faisalabad 2008) without dockage, was used. Before the trials, the moisture content of the grains was 12.0%, as calculated by a moisture meter (Dickey-John Multigrain CAC II; Dickey-John Co., Lawrence, KS, USA).

### 2.3. DE Formulation

The DE formulation (DEA) consisting of freshwater DE (90%) + 0.25% abamectin as an active ingredient (a.i.) was utilized at 35 ppm or 25 ppm for bioassays related to grains, and 2 gr/m^2^ for surface treatment, against larval and adult individuals of *T. castaneum* [77]. The origin of the DE is fresh water, and it is constituted by 89% amorphous silicon dioxide, along with 1.7% Fe_2_O_3_, 4.0% Al_2_O_3_, <1% K_2_O and MgO, 1.4% CaO, and 3% moisture. The median size of particles of the DE is 10 μm. It has a specific gravity (s.g.) of 2.2, a surface area of 35.7 m^2^/g, pH = 8, and 0.1% crystalline silica content [82], which is < the non-threatening level of 1% for human safety [83]. Abamectin consisted of a mixture of avermectins, specifically 80% B1a and 20% B1b avermectins, obtained from *Streptomyces avermitilis* (ex. Burg et al.) Kim and Godfellow (Actinomycetales: Streptomycetaceae).

### 2.4. Fungal Formulation

Two EPF isolates, namely, *B. bassiana* (WG-13) and *M. anisopliae* (WG-03) sourced from of the Microbial Control Laboratory, were used in the experimental assays. The isolates were preserved on Sabouraud Dextrose Agar (SDA) slopes in test tubes at 4 °C. Mass cultivation of the cultures was performed on Petri dishes (10 cm diameter) with Potato Dextrose Agar (PDA), incubated for 10 days at 25 °C and 14 h Light:10 h dark. Subsequently, using a sterile scalpel, the conidia were collected from the dishes and placed into a falcon tube (50 mL) containing 30 mL of a sterile solution of Tween 80 (0.05%) (Merck, Kenilworth, NJ, USA). Following this, the conidia suspension was subjected to agitation using a Vortex (Velp Scientifca Srl, Usmate Velate, Italy) for 5 min, together with eight sterile beads made of glass to facilitate the process. The concentration of both *B. bassiana* and *M. anisopliae* was standardized to 1.2 × 10^7^, 1.2 × 10^6^, and 1.2 × 10^5^ conidia/mL for each species of fungi, using a Neubauer-improved hemocytometer (Marienfeld, Lauda-Königshofen, Germany) and a microscope (BB.1152-PLi, Euromex Microscopen bv, Arnhem, The Netherlands). To estimate the germination of the conidia, two dishes (6 cm diameter) were prepared with a mixture of yeast (1%) and SDA. Each dish was inoculated with 0.1 mL of a solution that contained 1 × 10^6^ conidia/mL. The dishes were then sealed with parafilm and transferred into an incubator for 16 h at 25 °C under a 14:10 h (Light: Dark) cycle. Following this period, a non-contaminated cover slip was set on top of the dishes, and a total of 200 conidia were counted for each dish. Prior to each assay, the conidia germination was assessed under 400× magnification. It was found that >91% of both isolates had germinated.

### 2.5. Treatment of Wheat with a Single Method

A total of six treatments, plus the control, were executed for each developmental stage of *T. castaneum* (larvae and adults). A single dose rate of *B. bassiana* or *M. anisopliae* alone or combined with DEA were applied against larvae and adults of *T. castaneum*. Specifically, the treatments were *B. bassiana* alone at 1.2 × 10^5^ conidia/kg wheat, *M. anisopliae* alone at 1.2 × 10^5^ conidia/kg wheat, DEA at 35 ppm (35 mg DEA/kg wheat) alone, a combination of *B. bassiana* + DEA, a combination of *M. anisopliae* + DEA, and a combination of *B. bassiana* + *M. anisopliae* + DEA. Two additional kilograms of grains were left untreated to serve as control groups. One control group was used for larvae, and the other one was used for adults of *T. castaneum*. The treatment for the control was water that contained Tween 80 (0.05%) [77]. For each treatment, 1 kg lots were laid in slim layers on individual trays. The EPF were applied as liquids, while DEA was applied as dust. Spraying was conducted using a different airbrush for each treatment (Master Multipurpose Airbrush, San Diego, CA, USA). One milliliter of each conidial suspension or control was treated on 1 kg wheat. The treated grains were then conveyed into individual 3 L glass jars and shaken manually for 10 min to accomplish an equal distribution of conidia inside the mass of the grain. Considering the application of DEA, 1 kg of treated wheat was conveyed into a 3 L glass jar and shaken as aforementioned. For combined treatments, the aqueous conidial suspensions were applied first, followed by the DEA, conveyed to 3 L glass jars, and shaken as described above. Subsequently, three 60 g samples from each treated lot and the control were weighted using a balance (ELB 300 Shimadzu, Kyoto, Japan), conveyed into separate glass vials (diameter = 7 cm, height = 12 cm), and labeled appropriately. Thereafter, 60 individuals of *T. castaneum* were liberated in each vial and preserved at 30 °C and 65% RH. The lid of the vial carried a central hole 15 mm in diameter, which was cladded with gauze to facilitate adequate aeration within the vial. To prevent the escape of insects, the upper interior surfaces of the vials were smoothed using polytetrafluoroethylene (Sigma-Aldrich Chemie GmbH, Taufkirchen, Germany). New vials were prepared per exposure. Following the completion experimental period, the insects subjected to testing were removed from the wheat. Subsequently, the wheat was reintroduced into vials and repositioned in the incubators under the previously mentioned conditions for the documentation of offspring production. The mortality rate was estimated after 7, 14, and 21 days of exposure of the individuals to treated wheat samples, while the progeny was determined after 62 days [78]. The aforementioned protocol was conducted independently for each developmental stage of *T. castaneum* (adults of mixed sex, <14 days old, and larvae between third and fourth instar) and repeated three times, using a new series of vials for each replication (3 × 3 = 9 replications per treatment for each of the 6 treatments). The same procedure was replicated for each of the four populations studied in this investigation (Multan, Rawalpindi, Rahim Yar Khan, and Faisalabad). Progeny were adults and immatures of *T. castaneum*.

### 2.6. Treatment of Wheat with Different Methods

The treatments of fungi and DEA were applied to wheat (var. Faisalabad 2008) by two different application methods: dusting and spraying. Single and combined treatments are as mentioned above. The spraying of combined DEA and fungal conidia was carried out by preparing aqueous suspensions of DEA and each fungus at 1.2 × 10^7^ conidia/kg wheat. For the treatment of DEA alone, DEA at 25 mg was made up with water (distilled) to 1 mL. Spraying of the fungal suspensions was carried out as above. Spraying of DEA suspension was performed using a different airbrush (Master Multipurpose Airbrush, US Art Supply, San Diego, CA, USA). One milliliter of DEA suspension was applied on 1 kg wheat. The treated grains were then conveyed into individual 3 L glass jars and shaken manually for 10 min to accomplish an equal distribution of DEA particles into the mass of the grains. Concerning dusting, single and combined treatments were conducted as described above, with concentrations of the aqueous conidial suspensions at 1.2 × 10^7^ conidia/kg wheat and the DEA at 25 ppm. Fungal treatments were applied as liquids, while DEA was applied as dust, as described above. In addition, two lots containing 1 kg wheat were left untreated to serve as the control, one for each application method, as previously mentioned. The mortality rates were documented in the manner explained earlier, with the mortality rate assessed 10 days after the exposure of the insects to the sprayed or dusted wheat. The entire bioassay was replicated three times in total per population and per method, with each iteration involving the use of fresh insect individuals and wheat. This process was conducted independently for larvae and adults of *T. castaneum.*

### 2.7. Surface Treatment

For the surface treatment bioassays, three distinct surfaces were employed, namely, 1 mm thick galvanized steel (Pakistan Steel Mills Corporation, Karachi, Pakistan), approx. 40 mm thick concrete (D.G. Khan Cement, Lahore, Pakistan), and 250 GSM (g/m^2^) jute bags (Punjab Food Department, Faisalabad, Pakistan). The steel and jute bags were meticulously crafted to match the dimensions of the Petri dishes (diameter = 8 cm, height = 1.5 cm high, surface = 50.27 cm^2^). To prepare the concrete surface, a mixture of water and concrete (*f* = 0.4–0.6 w/c) was made into a slurry, which was subsequently poured into the dishes and allowed to dry for 24 h. Prior to the commencement of the experiment, surfaces were thoroughly cleaned to remove any debris and subsequently placed under experimental conditions at 30 °C and 65% RH. To impede insects from escaping from treated surfaces, sides of dishes were smoothed using polytetrafluoroethylene (Sigma-Aldrich Chemie GmbH, Taufkirchen, Germany).

For surface treatment, three dishes were prepared for each type of surface. Single and combined treatments were as mentioned above. The *B. bassiana* and *M. anisopliae* solutions were applied as liquids, while DEA was applied as dust. A total of 1 mL of each conidial suspension (*B. bassiana* and *M. anisopliae*: 1.2 × 10^6^ conidia/mL) was sprayed, using different airbrushes for each treatment, to three dishes for each type of surface. DEA (2 gr/m^2^ i.e., 0.0100 g/dish) [84] was sprinkled evenly over each substrate by sieving through a U.S. standard mesh sieve No 60 (0.250 mm mesh openings) (Advantech Manufacturing, Inc., New Berlin, WI, USA). For combined treatments, the spraying of conidia preceded the DEA application. Seven dishes (corresponding to six treatments and the control) for each type of surface were used. Thereafter, 10 larvae between the third and fourth instar obtained from the Faisalabad population were introduced to each dish and incubated at 30 °C and 65% RH. Mortality was assessed after 10 days of initiation. The above procedure was replicated three times, involving the use of fresh insect individuals and wheat. The bioassay was replicated three times in total (3 × 3). The above procedure was repeated for adults of mixed sex, <14 days old.

### 2.8. Data Analysis

Mortality was adjusted according to the formula of Abbott [85]. To normalize variance, the data were subjected to log(x + 1) transformation before analysis [86]. For the treatment of wheat with a single method, data were analyzed using a three-way ANOVA for each developmental stage of *T. castaneum*, with the population, concentration, and exposure as the main effects and mortality as the response variable. For the treatment of wheat with different methods, a three-way ANOVA was performed. The treatment, population, and method of application were the main effects, and mortality was the response variable. Concerning the surface treatment, a two-way ANOVA was conducted, with the dose and treatment as the main effects and mortality as the response variable. The data on the production of progeny were subjected to a two-way ANOVA, where the main effects were the treatment and species, and the response variable was the number of offspring produced. Data were analyzed separately for each developmental stage using the statistical software Minitab 13.2 (Minitab, 2002 Software Inc., Northampton, MA, USA). The test Tukey–Kramer (HSD) was utilized to separate means for mortality and offspring counts (*p* = 0.05) [87].

## 3. Results

### 3.1. Treatment of Wheat with a Single Method

The main effects and interactions affected larval and adult mortality significantly (Table 1). The mortality of both larvae and adults differed among treatments (*p* < 0.05) for all four tested populations. After a 7-day period of exposure, mortality was lower in single treatments (application of DEA and fungal isolates alone) compared to combined treatments. After exposure to *B. bassiana* + *M. anisopliae* + DEA, larval mortality ranged from 51.35% to 60.73%, and adult mortality ranged from 37.42% to 57.63% among the four populations (Table 2). At 14 days post-exposure, mortality was higher in combined treatments compared to single treatments. Concerning the combined treatment of *B. bassiana* + *M. anisopliae* + DEA, the Faisalabad and R.Y. Khan populations exhibited 100% larval mortality, followed by the Multan and Rawalpindi populations. Adult mortality ranged from 78.72% to 92.40% (Table 3). After the final assessment of 21 days, *B. bassiana* + DEA and *B. bassiana* + *M. anisopliae* + DEA treatments exhibited 100% larval mortality for all populations tested. The combination of *M. anisopliae* + DEA exhibited 100% mortality for all the populations except for the Rawalpindi population (96.58%). In the case of adult mortality, only *B. bassiana* + *M. anisopliae* + DEA revealed 100% mortality for all the populations (Table 4).

Concerning progeny, the main effects and interactions significantly affected the progeny emergence of *T. castaneum* in all four populations (Table 1). Significant differences were noted between all treatments applied compared to the control and all tested populations. Overall, the application of the combination of DEA and EPF resulted in lower progeny emergence compared to the application of either treatment alone (Table 5). The treatment of *B. bassiana* + *M. anisopliae* + DEA suppressed progeny emergence in all populations except for the Rawalpindi population, while progeny implementing *B. bassiana* + DEA was suppressed only in the Faisalabad and R.Y. Khan populations (Table 5).

### 3.2. Treatment of Wheat with Different Methods

Regarding the application method, the main effects and interactions for the mortality levels of larvae and adults were significant, apart from treatment × application methods for adults (Table 6). Upon the application of the treatments in wheat, there was a significant rise in the mortalities of both larvae and adults (following 10 days of exposure) through dusting compared to spraying. In the case of larvae, 100% larval mortality was observed for the Faisalabad and R.Y. Khan populations implementing *B. bassiana* + *M. anisopliae* + DEA with the dusting application method. The same combined method exhibited 100% larval mortality with the spraying application method only for the Faisalabad population. However, in the case of adult mortality, only the Faisalabad laboratory population exhibited 100% mortality for both dusting and spraying application methods implementing *B. bassiana* + *M. anisopliae* + DEA (Table 7).

### 3.3. Surface Treatment

Concerning surface treatment, the main effects and interactions on the mortality of larvae and adults were significantly affected by exposure (Table 8). A significant difference was observed in the mortality rates across all surfaces and treatments applied. Overall, all treatments were effective against larvae and adults of *T. castaneum* compared to the control. The combined treatments were more effective in comparison to a single application of DEA or EPF alone on all surfaces employed (Table 9). In particular, 100% larval mortality was observed for combined treatments of DEA and EPF on steel. Combined treatments of *B. bassiana* + DEA and *B. bassiana* + *M. anisopliae* + DEA exhibited 100% larval mortality on concrete. However, only the *B. bassiana* + *M. anisopliae* + DEA treatment was equally effective on jute bags (Table 9). Concerning adult mortality, only the *B. bassiana* + *M. anisopliae* + DEA treatment was 100% effective on all surfaces employed, apart from Jute bags (Table 9).

## 4. Discussion

Our findings indicate that longer exposures led to higher mortalities, particularly higher in *T. castaneum* larvae compared to adults. Additionally, the highest mortality rates were recorded in the Faisalabad population, followed by Rehaim Yar Khan, Rawalpindi, and Multan when treated with the combination of *B. bassiana* + *M. anisopliae* + DEA, as well as when treated with either treatment alone. After the final count, at 21 days of exposure, all populations tested under the *B. bassiana* + DEA, *M. anisopliae* + DEA, and *B. bassiana* + *M. anisopliae* + DEA treatments exhibited 100% larval mortality, apart from the Rawalpindi population, which exhibited 96.58% larval mortality under the *M. anisopliae* + DEA treatment. Regarding adult mortality, only *B. bassiana* + *M. anisopliae* + DEA exhibited 100% mortality for all populations.

The effectiveness of IPM programs depends on understanding the compatibility of EPF with pesticides used to protect crops [88,89]. The maximum mortality incurred due to additive effects between DEs and EPF is poorly understood. However, Dal Bello et al. [13] exhibited that DE and *B. bassiana* work jointly to combat various pests of stored grains, with no adverse effects on the EPF’s germination. Furthermore, previous research highlighted that DE increases the effectiveness of *B. bassiana* against larvae of *T. castaneum*, particularly due to the abrasive action of DE on the insect’s cuticle [51,53]. This leads to cuticle desiccation, thus facilitating the conidial adhesion, germination, and conidial penetration of the fungus inside the insect [53]. Concerning the combination of *B. bassiana* + *M. anisopliae*, it has been documented that these two EPF species have been more effective against *Amblyomma variegatum* (F.) (Ixodida: Ixodidae) as a combination rather than alone [90]. The EPF *B. bassiana* and *M. anisopliae* can exhibit an enhanced effect against pests, as demonstrated in this investigation and previous research. This effect appears to be further elevated when applied in combination with DE, due to its ability to damage the insect’s cuticle. However, further research is required to fully understand the mechanism behind this combined effect and to optimize the application of this combination treatment for sustainable and effective pest management.

Former research studies have highlighted the additive effect of mixed applications of *B*. *bassiana* with DE on stored-product pests, such as *Callosobruchus maculatus* (F.) (Coleoptera: Chrysomelidae), *Oryzaephilus surinamensis* (L.) (Coleoptera: Silvanidae) [91], *Rhyzopertha dominica* (F.) (Coleoptera: Bostrychidae), *Cryptolestes ferrugineus* (Stephens) (Coleoptera: Laemophloeidae) [71,92], *Liposcelis paeta* Pearman (Psocoptera: Liposcelididae) [71], and *Sitophilus oryzae* (L.) (Coleoptera: Curculionidae) [93]. The combined use of *M. anisopliae* and DE has also been subject to investigation against several pests, such as *Plodia interpunctella* (Hübner) (Lepidoptera: Pyralidae) [94], *L. paeta*, *C. ferrugineus*, and *R. dominica* [55]. By investigating the efficacy of *B. bassiana* + DE, *M. anisopliae* + DE, and fungi alone on *T. castaneum* adults, Shafighi et al. [79] exhibited that the highest mortalities were recorded when the combined treatments were applied with an exposure interval of 7 days after application, as opposed to the use of EPF alone. Likewise, recent studies emphasized the enhanced effects of the combined applications of DEs with either *B. bassiana* [77,78] or *M. anisopliae* [55,79] against *T. castaneum*, in a manner corresponding to our results. For example, the combined application of Grain-Guard, an enhanced DE with 0.25% abamectin, with *M*. *anisopliae* has been reported to elevate the EPF impact on several stored-product pests, including *T. castaneum* [55], as demonstrated in our research as well.

Concerning progeny production, a significant effect on the progeny of *T. castaneum* was observed in all treatments compared to the control, with the combined treatments demonstrating greater efficacy than single treatments. Furthermore, the combined treatment of DEA and both EPF species suppressed the progeny production during a period of 21 days in all populations, apart from the Rawalpindi population. Rizwan et al. [51] observed reduced progeny production in *T. castaneum* with the combined application of high concentrations of *B. bassiana* and DE formulation Dafil 610. According to our findings, the combination of enhanced DE with abamectin and single or both EPF was found to be virulent against *T. castaneum*. The reduction in insect progeny production is a critical parameter in stored-product protection, as it serves to decrease grain damage in the absence of a parental adult population [95,96].

Previous research has indicated that the effectiveness of EPF against insect pests is influenced by various factors, including the abiotic conditions, the virulence of the specific EPF species or strain, the type of grain, and the susceptibility of the target species/instar. For example, the mortality rate of *T. castaneum* adults treated with *B. bassiana* was found to be higher at 30 °C compared to 25 °C or 20 °C [45]. Commercial formulations of *B*. *bassiana* (Naturalis-L), *Verticillium lecanii* (Mycotal), and *M*. *anisopliae* (Met-52), evaluated against adults and larval instars of *T*. *castaneum*, have highlighted *B*. *bassiana* and *M. anisopliae* as more virulent than *V. lecanii* against this pest [97]. Likewise, Wakil et al. [98] demonstrated significant differences in the mortalities of *T. castaneum* when exposed to four isolates of *B. bassiana* and three isolates of *M. anisopliae*. The observed differences were significant at both species and isolate levels. The host specificity of EPF varies substantially both among and within genera. While many EPF species are highly specific to certain insect hosts, others, such as *M. anisopliae* and *B. bassiana*, have been widely studied, having a broad range of hosts [99]. Concerning the abiotic factors affecting the fungal virulence, the grain type has been identified as a highly influential factor. In fact, the type of grain can play an equally important role as other abiotic factors in determining the virulence of EPF [100]. The susceptibility of different instars of a particular species to EPF is known to vary, as observed for *T. castaneum*. For instance, Baek et al. [101] demonstrated that a higher mortality rate was recorded in larvae of *T. castaneum* compared to adults after 72 h of exposure to a *B. bassiana* isolate. Likewise, our results document higher larval susceptibility to combined treatments compared to adults. In the realm of EPF, virulence is predicated upon the attachment of conidia to the body of the host, their subsequent germination, and, ultimately, their penetration into the insect [42,43]. The variable virulence of EPF may be partially explained by the fact that insect stages possess different cuticular layers, which vary in the morphology, softness, and thickness of the epicuticle [102,103], as suggested in previous research on the susceptibility of *T. castaneum* to EPF [77,104].

Selective application methods, such as dusting, slurries, or liquid aqueous spraying, have been employed to manage stored-grain insect pests [67]. Fields and Korunić [105] reported that dust application is more effective than aqueous spray, in line with Athanassiou et al. [106], who also found that DE applied as dust was more efficient than spraying against *T. confusum* and *S. oryzae*. It is worth noting that the efficacy of DE Perma-Guard was reduced when the moisture content increased to 14% against three stored-grain beetles [107]. Admixing dry conidia of EPF with dusts such as DE enhanced their efficacy in both laboratory and storage conditions [72,108,109,110]. In addition, the use of liquid formulations of EPF, where conidia are suspended in oily liquid ingredients, was successful; however, little is known about the success of this combination in storage environments [110]. In our study, dusting was more efficient than spraying in larvae and adults of *T. castaneum* when implementing *B. bassiana* + *M. anisopliae* + DEA treatments, further confirming previous research on the efficacy of different application methods applying DE. Nevertheless, the efficacy of ten different isolates of EPF against *R. dominica* was higher when applied through dusting rather than spraying [111], further supporting our results.

The grain commodities are stored in different storage facilities all over the world. In Pakistan, various types of storage structures are employed to store wheat commodities, such as house-type warehouses commonly known as “godowns”, bunkers, hexagonal bins, binishells, steel/concrete silos, and temporary open storage bags made of polyethylene, jute, and hessian (“ganjis”) [71,112]. On the basis of the findings of this research, the combined use of EPF with DEs as structural treatments offers an alternative approach for the management of noxious insects, taking into account that their mortalities may vary among different types of surfaces. Among the three tested surfaces, higher mortality was observed when treatments were applied on steel, followed by concrete and jute bags. Previously, Arthur [113] found that *T*. *castaneum* was susceptible to deltamethrin dusts applied on concrete, tile, and wood surfaces, while in latter research, the author found that the survival rate of *T*. *castaneum* was lower on concrete than on plywood or tile surfaces when treated with the insecticidal pyrrole chlorfenapyr [114]. The efficacy of four organophosphates, which included pirimiphos-methyl, was found to persist longer on galvanized steel compared to concrete when tested against three species of psocids [115]. By studying the effectiveness of malathion on several storage bag fabrics, Paudyal et al. [116] observed higher mortality and lower progeny production of *T. castaneum* on polypropylene bags rather than any other absorbent fabric, including jute bags. The effectiveness of a treatment on different surfaces can be attributed to the physical nature of porosity. Porous surfaces, such as concrete, wood, and fabric, exhibit a lower residual efficacy compared to non-porous surfaces (e.g., metal, tile, glass), directly affecting the insect’s insecticide uptake [114,117]. This stresses the necessity for treatments to be tested on a wide range of surfaces to account for the potential variations in efficacy that may arise due to the surface type, pest species, and insecticide formulation.

## 5. Conclusions

The present study has demonstrated that biological insecticides provide effective control in storage conditions as compared to chemical insecticides. This scenario represents a more practical option akin to real-world field conditions. Biological insecticides exhibit greater efficacy than chemical insecticides when used at doses compatible with IPM practices. Our results imply that combining *M. anisopliae* and *B. bassiana* with DEA can be an effective strategy for managing populations of *T. castaneum*. The study also highlights that EPF and DEA enhanced with abamectin offer long-term and enhanced management of *T. castaneum*, inhibiting the pest’s ability to reproduce effectively.

## Figures and Tables

**Table 1:** ANOVA parameters for the larval and adult mortality and progeny emergence of four populations of *T. castaneum* on wheat treated with *B. bassiana*, *M. anisopliae*, and DEA (for mortality total DF = 755; for progeny total DF = 287).

Effect	DF	Larva		Adult	
		*F*	*p*	*F*	*p*
Mortality					
Population	3	448.46	<0.01	774.59	<0.01
Dose	6	2922.01	<0.01	5926.11	<0.01
Interval	2	18,750.7	<0.01	29,245.5	<0.01
Population × dose	18	3.48	<0.01	11.79	<0.01
Population × interval	6	18.33	<0.01	28.23	<0.01
Dose × interval	12	224.74	<0.01	342.69	<0.01
Population × dose × interval	36	7.87	<0.01	12.30	<0.01
Progeny					
Population	3	8559.21	<0.01	11,921.80	<0.01
Treatment	7	92.08	<0.01	195.96	<0.01
Population × treatment	21	12.39	<0.01	12.89	<0.01

**Table 2:** Mean mortality (% ±SE) of larvae and adults of four populations of *T. castaneum* after a 7-day exposure on wheat treated with *B. bassiana* (WG-13 at 1.2 × 10^5^ conidia/kg of wheat), *M. anisopliae* (WG-03 at 1.2 × 10^5^ conidia/kg of wheat), and DEA (35 ppm) alone or in combinations. Means followed by the same upper-case letters within each column are not significantly different (in all cases, DF = 6, 62, Tukey–Kramer (HSD) test at *p* = 0.05). Means followed by the same lower-case letters within each row are not significantly different (in all cases, DF = 3, 35, Tukey–Kramer (HSD) test at *p* = 0.05).

Host Stage	Treatment	Populations					
		Faisalabad	R.Y. Khan	Rawalpindi	Multan	*F*	*p*
Larva	*Bb*	12.49 ± 1.05 Da	10.46 ± 0.55 Ea	7.20 ± 0.98 Db	10.45 ± 0.70 Eab	6.63	˂0.01
	*Ma*	10.41 ± 0.98 Da	9.29 ± 0.98 Ea	7.59 ± 0.77 Da	8.15 ± 0.76 Ea	2.00	0.133
	DEA	28.26 ± 1.21 Ca	26.06 ± 1.29 CDa	19.03 ± 1.05 Bb	21.29 ± 0.85 CDb	14.5	˂0.01
	*Bb* + *Ma*	23.88 ± 1.39 Ca	20.70 ± 1.06 Dab	13.69 ± 1.07 Cc	16.50 ± 1.29 Dbc	13.8	˂0.01
	*Bb* + DEA	38.14 ± 1.41 Ba	32.67 ± 1.30 Bb	23.81 ± 1.79 Bc	27.77 ± 1.38 Bc	32.2	˂0.01
	*Ma* + DEA	36.59 ± 1.39 Ba	31.31 ± 1.46 BCb	22.48 ± 1.70 Bc	26.06 ± 1.27 BCc	35.9	˂0.01
	*Bb* + *Ma* + DEA	60.73 ± 1.14 Aa	54.37 ± 1.84 Ab	49.35 ± 0.95 Ab	51.35 ± 1.84 Ab	12.7	˂0.01
	*F*	436	418	339	324		
	*p*	˂0.01	˂0.01	˂0.01	˂0.01		
Adult	*Bb*	8.63 ± 0.88 Ea	6.80 ± 0.40 Eab	3.20 ± 0.71 Ec	5.62 ± 0.77 Ebc	10.0	˂0.01
	*Ma*	7.88 ± 1.20 Ea	6.61 ± 0.51 Eab	2.26 ± 0.49 Ec	4.30 ± 0.92 Ebc	10.9	˂0.01
	DEA	21.06 ± 1.03 Ca	18.87 ± 1.12 Cab	14.55 ± 1.28 Cc	16.36 ± 1.12 Cbc	11.9	˂0.01
	*Bb* + *Ma*	15.80 ± 0.71 Da	10.75 ± 0.82 Db	6.61 ± 0.65 Dc	9.38 ± 0.94 Dbc	23.6	˂0.01
	*Bb* + DEA	33.49 ± 1.36 Ba	28.55 ± 1.13 Bb	21.31 ± 0.84 Bd	27.30 ± 1.68 Bb	24.9	˂0.01
	*Ma* + DEA	31.82 ± 1.11 Ba	27.20 ± 1.31 Bb	18.64 ± 0.90 Bd	25.79 ± 1.76 Bb	24.7	˂0.01
	*Bb* + *Ma* + DEA	57.63 ± 0.82 Aa	52.15 ± 0.98 Ab	37.42 ± 1.81 Ad	45.74 ± 1.85 Ac	101	˂0.01
	*F*	436	418	339	324		
	*p*	˂0.01	˂0.01	˂0.01	˂0.01		

**Table 3:** Mean mortality (% ±SE) of larvae and adults of four populations of *T. castaneum* after a 14-day exposure on wheat treated with *B. bassiana* (WG-13 at 1.2 × 10^5^ conidia/kg of wheat), *M. anisopliae* (WG-03 at 1.2 × 10^5^ conidia/kg of wheat), and DEA (35 ppm) alone or in combinations. Means followed by the same upper-case letters within each column are not significantly different (in all cases, DF = 6, 62, Tukey–Kramer (HSD) test at *p* = 0.05). Means followed by the same lower-case letters within each row are not significantly different (in all cases, DF = 3, 35, Tukey–Kramer (HSD) test at *p* = 0.05).

Host Stage	Treatment	Populations					
		Faisalabad	R.Y. Khan	Rawalpindi	Multan	*F*	*p*
Larva	*Bb*	27.24 ± 1.45 Da	24.04 ± 0.66 Da	16.82 ± 1.22 Fc	21.95 ± 1.15 Db	14.3	˂0.01
	*Ma*	24.04 ± 0.66 Da	22.51 ± 0.85 DAa	12.85 ± 0.91 Fc	17.78 ± 1.17 Db	20.0	˂0.01
	DEA	55.14 ± 1.20 Ca	51.33 ± 1.37 Ca	41.27 ± 1.10 Dc	46.20 ± 1.30 Cb	23.3	˂0.01
	*Bb* + *Ma*	53.44 ± 1.26 Ca	48.30 ± 1.26 Cb	31.60 ± 1.23 Ed	41.65 ± 1.11 Cc	59.6	˂0.01
	*Bb* + DEA	81.63 ± 2.21 Ba	73.09 ± 1.34 Bb	60.20 ± 1.42 Bc	67.39 ± 1.47 Bb	30.1	˂0.01
	*Ma* + DEA	76.32 ± 1.51 Ba	68.61 ± 1.86 Bb	51.67 ± 1.61 Cd	62.52 ± 1.08 Bc	45.4	˂0.01
	*Bb* + *Ma* + DEA	100.0 ± 0.00 Aa	100.0 ± 0.00 Aa	82.37 ± 1.58 Ac	93.19 ± 1.19 Ab	70.3	˂0.01
	*F*	956	729	754	710		
	*p*	˂0.01	˂0.01	˂0.01	˂0.01		
Adult	*Bb*	20.78 ± 0.86 Fa	15.67 ± 1.18 Fb	9.27 ± 0.77 Fc	13.29 ± 0.71 Fb	28.2	˂0.01
	*Ma*	19.63 ± 1.01 Fa	16.63 ± 1.05 Fa	12.12 ± 0.82 Fb	13.62 ± 0.71 Fb	18.5	˂0.01
	DEA	48.60 ± 1.56 Da	40.68 ± 1.07 Db	27.88 ± 1.23 Dd	34.58 ± 1.04 Dc	95.9	˂0.01
	*Bb* + *Ma*	31.56 ± 0.72 Ea	29.31 ± 1.05 Ea	16.67 ± 0.91 Ec	23.18 ± 0.91 Eb	54.2	˂0.01
	*Bb* + DEA	74.46 ± 1.04 Ba	71.36 ± 1.09 Ba	49.14 ± 1.77 Bc	58.54 ± 1.11 Bb	134	˂0.01
	*Ma* + DEA	69.73 ± 1.09 Ca	62.09 ± 1.14 Cb	41.70 ± 1.09 Cd	52.63 ± 1.03 Cc	123	˂0.01
	*Bb* + *Ma* + DEA	92.40 ± 1.05 Aa	87.86 ± 1.16 Ab	78.72 ± 1.00 Ac	85.17 ± 1.32 Ab	25.0	˂0.01
	*F*	956	729	754	710		
	*p*	˂0.01	˂0.01	˂0.01	˂0.01		

**Table 4:** Mean mortality (% ±SE) of larvae and adults of four populations of *T. castaneum* after a 21-day exposure on wheat treated with *B. bassiana* (WG-13 at 1.2 × 10^5^ conidia/kg of wheat), *M. anisopliae* (WG-03 at 1.2 × 10^5^ conidia/kg of wheat), and DEA (35 ppm) alone or in combinations. Means followed by the same upper-case letters within each column are not significantly different (in all cases, DF = 6, 62, Tukey–Kramer (HSD) test at *p* = 0.05). Means followed by the same lower-case letters within each row are not significantly different (in all cases, DF = 3, 35, Tukey–Kramer test (HSD) test at *p* = 0.05). Where dashes exist, no analysis was performed.

Host Stage	Treatment	Populations					
		Faisalabad	R.Y. Khan	Rawalpindi	Multan	*F*	*p*
Larva	*Bb*	75.31 ± 1.62 Ca	68.72 ± 1.51 Db	57.38 ± 1.36 Dc	65.48 ± 1.20 Db	26.9	˂0.01
	*Ma*	71.89 ± 1.63 Ca	65.68 ± 1.86 Dab	54.12 ± 1.41 Dc	64.82 ± 1.66 Db	20.0	˂0.01
	DEA	84.34 ± 1.71 Ba	83.56 ± 1.33 Ca	73.78 ± 1.68 Cb	80.69 ± 1.67 Ca	8.94	˂0.01
	*Bb* + *Ma*	100.0 ± 0.00 Aa	92.69 ± 1.72 Bb	84.11 ± 1.26 Bc	89.46 ± 2.09 Bbc	19.7	˂0.01
	*Bb* + DEA	100.0 ± 0.00 Aa	100.0 ± 0.00 Aa	100.0 ± 0.00 Aa	100.0 ± 0.00 Aa	0.00	˂0.01
	*Ma* + DEA	100.0 ± 0.00 Aa	100.0 ± 0.00 Aa	96.58 ± 1.26 Ab	100.0 ± 0.00 Aa	7.32	˂0.01
	*Bb* + *Ma* + DEA	100.0 ± 0.00 Aa	100.0 ± 0.00 Aa	100.0 ± 0.00 Aa	100.0 ± 0.00 Aa	-	-
	*F*	138	148	148	153		
	*p*	˂0.01	˂0.01	˂0.01	˂0.01		
Adult	*Bb*	61.37 ± 1.54 Da	56.47 ± 1.37 Eb	49.53 ± 1.03 Ec	51.16 ± 0.93 Fc	30.7	˂0.01
	*Ma*	57.54 ± 1.92 Da	54.39 ± 1.96 Ea	44.35 ± 0.91 Fc	50.58 ± 1.83 Fb	38.9	˂0.01
	DEA	76.67 ± 1.11 Ca	71.84 ± 1.12 Eb	65.18 ± 1.86 Dc	70.60 ± 1.56 Db	21.2	˂0.01
	*Bb* + *Ma*	89.30 ± 0.96 Ba	83.56 ± 1.14 Cb	78.59 ± 1.10 Cc	82.58 ± 1.97 Dbc	17.8	˂0.01
	*Bb* + DEA	100.0 ± 0.00 Aa	98.86 ± 0.94 Aa	83.75 ± 1.04 Bc	92.51 ± 0.91 Bb	50.9	˂0.01
	*Ma* + DEA	100.0 ± 0.00 Aa	91.41 ± 0.99 Bb	81.26 ± 0.96 BCc	88.09 ± 1.05 Cb	91.3	˂0.01
	*Bb* + *Ma* + DEA	100.0 ± 0.00 Aa	100.0 ± 0.00 Aa	100.0 ± 0.00 Aa	100.0 ± 0.00 Aa	-	-
	*F*	388	380	413	415		
	*p*	˂0.01	˂0.01	˂0.01	˂0.01		

**Table 5:** Mean number (±SE) of four populations of *T. castaneum* individuals per vial, after a 62-day exposure interval on wheat treated with *B. bassiana* (WG-13 at 1.2 × 10^5^ conidia/kg of grain), *M. anisopliae* (WG-03 at 1.2 × 10^5^ conidia/kg of grain), and DEA (35 ppm) alone or in combinations. Means followed by the same upper-case letters within each column are not significantly different (in all cases, DF = 7, 71, Tukey–Kramer (HSD) test at *p* = 0.05). Means followed by the same lower-case letters within each row are not significantly different (in all cases, DF = 3, 35, Tukey–Kramer (HSD) test at *p* = 0.05).

Treatment	Populations					
	Faisalabad	R.Y. Khan	Rawalpindi	Multan	*F*	*p*
*Bb*	29.2 ± 0.46 Cd	31.3 ± 0.48 Cc	38.2 ± 0.46 Ca	34.2 ± 0.54 Cb	67.7	˂0.01
*Ma*	32.8 ± 0.53 Bd	35.1 ± 0.51 Bc	43.2 ± 0.53 Ba	39.2 ± 0.46 Bb	79.4	˂0.01
DEA	19.7 ± 0.45 Dc	21.1 ± 0.51 Db	27.7 ± 0.46 Da	23.1 ± 0.50 Db	45.2	˂0.01
*Bb* + Ma	8.5 ± 0.37 Ec	9.3 ± 0.41 Ec	13.3 ± 0.44 Ea	11.4 ± 0.39 Eb	28.8	˂0.01
*Bb* + DEA	0.0 ± 0.00 Fc	0.0 ± 0.00 Gc	9.3 ± 0.40 Fa	4.4 ± 0.38 Fb	89.5	˂0.01
*Ma* + DEA	1.1 ± 0.26 Fc	5.5 ± 0.37 Fb	9.5 ± 0.34 Fa	6.4 ± 0.37 Fb	291	˂0.01
*Bb* + *Ma* + DEA	0.0 ± 0.00 Fb	0.0 ± 0.00 Gb	1.2 ± 0.27 Ga	0.0 ± 0.00 Gb	19.4	˂0.01
Control	92.0 ± 1.36 Aa	89.1 ± 1.19 Aa	91.7 ± 1.22 Aa	91.6 ± 1.04 Aa	1.26	0.30
*F*	2722	3462	2480	2591		
*p*	˂0.01	˂0.01	˂0.01	˂0.01		

**Table 6:** ANOVA parameters for the larval and adult mortality of four populations of *T. castaneum* using two application methods on wheat treated with *B. bassiana*, *M. anisopliae*, and DEA (total DF = 503).

Effect	DF	Larva		Adult	
		*F*	*p*	*F*	*p*
Treatment	6	2175.0	<0.01	2145.88	<0.01
Population	3	281.0	<0.01	355.31	<0.01
Method	1	367.3	<0.01	424.78	<0.01
Treatment × population	18	6.18	<0.01	10.85	<0.01
Treatment × method	6	333.22	<0.01	0.73	0.53
Population × method	3	3.00	0.03	271.51	<0.01
Treatment × population × method	18	6.72	<0.01	3.49	<0.01

**Table 7:** Mean mortality (% ±SE) of larvae and adults of four populations of *T. castaneum* after a 10-day exposure on wheat treated with *B. bassiana* (WG-13 at 1.2 × 10^7^ conidia/kg of wheat), *M. anisopliae* (WG-03 at 1.2 × 10^7^ conidia/kg of wheat), and DEA (25 ppm) alone or in combinations, using two application methods. Means followed by the same upper-case letters within each column are not significantly different (in all cases, DF = 6, 62, Tukey–Kramer (HSD) test at *p* = 0.05). Means followed by the same lower-case letters within each row are not significantly different (in all cases, DF = 3, 35, Tukey–Kramer (HSD) test at *p* = 0.05).

Application Methods	Insect Stage	Treatment	Faisalabad	R.Y. Khan	Rawalpindi	Multan	*F*	*p*
Dusting	Larva	*Bb*	32.13 ± 2.11 Da	27.61 ± 0.85 EFab	21.36 ± 0.80 EFc	23.12 ± 1.27 Ebc	12.5	˂0.01
		*Ma*	25.69 ± 1.88 Da	22.68 ± 1.09 Fab	18.51 ± 0.93 Fb	19.74 ± 0.77 Eb	6.64	˂0.01
		DEA	46.58 ± 1.28 Ca	39.43 ± 2.09 Db	32.79 ± 1.79 Dc	35.27 ± 1.65 Dbc	12.2	˂0.01
		*Bb* + *Ma*	40.85 ± 1.98 Ca	33.84 ± 1.60 Deb	27.28 ± 1.12 DEc	31.49 ± 1.52 Dbc	12.8	˂0.01
		*Bb* + DEA	84.44 ± 1.62 Ba	76.63 ± 1.84 Bb	58.57 ± 1.99 Bc	70.54 ± 1.95 Bb	34.5	˂0.01
		*Ma* + DEA	79.20 ± 1.72 Ba	62.78 ± 1.99 Cb	51.75 ± 1.40 Cc	57.85 ± 1.90 Cbc	44.2	˂0.01
		*Bb* + *Ma* + DEA	100.0 ± 0.00 Aa	100.0 ± 0.00 Aa	89.32 ± 1.53 Ab	98.28 ± 0.75 Aa	35.8	˂0.01
		*F*	307	329	317	415		
		*p*	˂0.01	˂0.01	˂0.01	˂0.01		
	Adult	*Bb*	28.85 ± 0.74 EFa	23.58 ± 1.05 EFb	17.88 ± 0.97 EFc	21.47 ± 1.12 Ebc	21.7	˂0.01
		*Ma*	22.73 ± 1.23 Fa	19.36 ± 1.22 Fab	15.57 ± 1.21 Fb	17.68 ± 1.19 Eb	6.16	˂0.01
		DEA	39.30 ± 1.94 Da	32.07 ± 2.02 Db	26.62 ± 0.93 Db	29.27 ± 1.26 Db	11.5	˂0.01
		*Bb* + *Ma*	34.53 ± 1.48 DEa	28.30 ± 1.23 Deb	22.44 ± 0.80 Ec	23.37 ± 0.81 DEc	24.4	˂0.01
		*Bb* + DEA	71.68 ± 1.95 Ba	59.35 ± 1.67 Bb	47.31 ± 1.58 Bc	52.65 ± 1.53 Bc	38.5	˂0.01
		*Ma* + DEA	58.81 ± 1.85 Ca	50.59 ± 1.59 Cb	39.70 ± 1.41 Cc	45.79 ± 1.74 Cbc	23.6	˂0.01
		*Bb* + *Ma* + DEA	100.0 ± 0.00 Aa	96.18 ± 1.11 Aa	75.48 ± 2.02 Ac	87.66 ± 1.53 Ab	61.5	˂0.01
		*F*	349	348	250	344		
		*p*	˂0.01	˂0.01	˂0.01	˂0.01		
Spraying	Larva	*Bb*	39.59 ± 1.17 Da	34.22 ± 1.57 DEb	27.10 ± 1.5 DEc	31.72 ± 1.35 Dbc	15.4	˂0.01
		*Ma*	33.73 ± 1.50 Ea	29.67 ± 1.65 Eab	23.51 ± 0.74 Ec	27.40 ± 1.34 Dbc	9.95	˂0.01
		DEA	19.67 ± 1.02 Fa	16.54 ± 1.02 Fab	12.70 ± 0.73 Fc	15.57 ± 0.79 Ebc	10.2	˂0.01
		*Bb* + *Ma*	65.88 ± 1.24 Ba	54.32 ± 1.43 Bb	39.08 ± 1.36 Bd	47.72 ± 1.88 Bc	56.6	˂0.01
		*Bb* + DEA	54.01 ± 1.27 Ca	46.82 ± 2.00 Cb	35.68 ± 1.48 BCc	39.67 ± 2.10 Cc	21.3	˂0.01
		*Ma* + DEA	41.50 ± 0.88 Da	39.40 ± 1.21 Da	30.31 ± 1.90 CDb	33.05 ± 1.88 CDb	11.7	˂0.01
		*Bb* + *Ma* + DEA	100.0 ± 0.00 Aa	96.76 ± 1.01 Aa	77.28 ± 2.00 Ac	85.56 ± 1.62 Ab	56.6	˂0.01
		*F*	557	311	208	190		
		*p*	˂0.01	˂0.01	˂0.01	˂0.01		
	Adult	*Bb*	35.58 ± 1.26 CDa	29.44 ± 1.49 CDb	23.29 ± 1.00 CDc	25.57 ± 0.75 Cbc	21.4	˂0.01
		*Ma*	31.64 ± 0.89 Da	26.62 ± 1.00 Db	20.44 ± 1.05 Dc	22.89 ± 1.11 Cbc	22.8	˂0.01
		DEA	15.33 ± 0.38 Ea	11.85 ± 1.10 Eb	7.73 ± 0.84 Ec	10.77 ± 0.75 Dbc	14.9	˂0.01
		*Bb* + *Ma*	51.49 ± 1.45 Ba	40.03 ± 1.87 Bb	32.56 ± 1.31 Bc	36.20 ± 1.28 Bbc	29.9	˂0.01
		*Bb* + DEA	39.93 ± 1.49 Ca	33.25 ± 1.45 Cb	25.73 ± 1.30 Cc	28.57 ± 1.74 Cbc	16.9	˂0.01
		*Ma* + DEA	34.80 ± 1.51 Da	27.92 ± 1.74 CDb	19.48 ± 1.13 Dc	23.85 ± 1.05 Cbc	21.9	˂0.01
		*Bb* + *Ma* + DEA	100.0 ± 0.00 Aa	82.56 ± 1.77 Ab	64.05 ± 1.65 Ac	76.90 ± 2.06 Ab	87.8	˂0.01
		*F*	551	213	214	252		
		*p*	˂0.01	˂0.01	˂0.01	˂0.01		

**Table 8:** ANOVA parameters for larval and adult mortality of *T. castaneum* on three surfaces treated with *B. bassiana*, *M. anisopliae*, and DEA (total DF = 188).

Effect	DF	Larva		Adult	
		*F*	*p*	*F*	*p*
Dose	6	2808.97	<0.01	2089	<0.01
Surface	2	92.22	<0.01	64.02	<0.01
Dose × surface	12	8.07	<0.01	2.22	0.01

**Table 9:** Mean mortality (% ±SE) of the Faisalabad population larvae and adults of *T. castaneum* after a 10-day exposure on three surface types treated with *B. bassiana* (WG-13 at 1.2 × 10^6^ conidia/kg of wheat), *M. anisopliae* (WG-03 at 1.2 × 10^6^ conidia/kg of wheat), and DEA (2 g/m^2^) alone or in combinations. Means followed by the same upper-case letters within each column are not significantly different (in all cases, DF = 6, 62, Tukey–Kramer (HSD) test at *p* = 0.05). Means followed by the same lower-case letters within each row are not significantly different (in all cases, DF = 2, 26, Tukey–Kramer (HSD) test at *p* = 0.05). Where dashes exist, no analysis was performed.

Host Stage	Treatment	Steel	Concrete	Jute Bag	*F*	*p*
Larva	*Bb*	24.60 ± 0.92 Da	22.45 ± 0.83 Da	19.17 ± 0.70 Fb	10.9	˂0.01
	*Ma*	21.56 ± 1.02 Da	19.03 ± 0.96 Dab	17.45 ± 1.09 Fb	4.05	˂0.01
	DEA	34.27 ± 1.32 Ca	28.55 ± 1.52 Cb	25.98 ± 0.99 Eb	10.7	˂0.01
	*Bb* + *Ma*	90.75 ± 1.49 Ba	85.17 ± 2.12 Ba	75.34 ± 2.04 Db	16.7	˂0.01
	*Bb* + DEA	100.0 ± 0.00 Aa	100.0 ± 0.00 Aa	89.58 ± 1.92 Bb	29.4	˂0.01
	*Ma* + DEA	100.0 ± 0.00 Aa	96.37 ± 1.23 Aa	82.40 ± 2.44 Cb	34.7	˂0.01
	*Bb* + *Ma* + DEA	100.0 ± 0.00 Aa	100.0 ± 0.00 Aa	100.0 ± 0.00 Aa	-	-
	*F*	1735	1065	552		
	*p*	˂0.01	˂0.01	˂0.01		
Adult	*Bb*	21.39 ± 1.24 Ea	18.65 ± 1.21 EFab	16.82 ± 1.00 DEb	3.95	˂0.01
	*Ma*	17.78 ± 0.90 Ea	14.88 ± 1.15 Fab	12.25 ± 1.11 Eb	6.77	˂0.01
	DEA	28.96 ± 1.50 Da	23.95 ± 1.11 Eb	21.73 ± 0.84 Db	9.73	˂0.01
	*Bb* + *Ma*	76.74 ± 1.79 Ca	71.67 ± 1.80 Da	62.76 ± 1.72 Cb	15.9	˂0.01
	*Bb* + DEA	95.27 ± 0.96 ABa	92.23 ± 1.55 Ba	85.24 ± 2.23 Bb	9.50	˂0.01
	*Ma* + DEA	91.63 ± 1.54 Ba	83.58 ± 1.99 Cb	80.92 ± 1.81 Bb	9.66	˂0.01
	*Bb* + *Ma* + DEA	100.0 ± 0.00 Aa	100.0 ± 0.00 Aa	93.39 ± 1.21 Ab	29.4	˂0.01
	*F*	870	710	556		
	*p*	˂0.01	˂0.01	˂0.01		

## Data Availability

Data are available within the article.
